# Inflammatory Cell Recruitment in Cardiovascular Disease

**DOI:** 10.3389/fcell.2021.635527

**Published:** 2021-02-18

**Authors:** Timoteo Marchini, Lucía Sol Mitre, Dennis Wolf

**Affiliations:** ^1^Department of Cardiology and Angiology I, University Heart Center Freiburg, Freiburg, Germany; ^2^Faculty of Medicine, University of Freiburg, Freiburg, Germany; ^3^Facultad de Farmacia y Bioquímica, Instituto de Bioquímica y Medicina Molecular (IBIMOL), Universidad de Buenos Aires, CONICET, Buenos Aires, Argentina

**Keywords:** atherosclerosis, myocardial infarction, recruitment, leukocyte, selectin, integrin, cytokine, chemokine

## Abstract

Atherosclerosis, the main underlying pathology for myocardial infarction and stroke, is a chronic inflammatory disease of middle-sized to large arteries that is initiated and maintained by leukocytes infiltrating into the subendothelial space. It is now clear that the accumulation of pro-inflammatory leukocytes drives progression of atherosclerosis, its clinical complications, and directly modulates tissue-healing in the infarcted heart after myocardial infarction. This inflammatory response is orchestrated by multiple soluble mediators that enhance inflammation systemically and locally, as well as by a multitude of partially tissue-specific molecules that regulate homing, adhesion, and transmigration of leukocytes. While numerous experimental studies in the mouse have refined our understanding of leukocyte accumulation from a conceptual perspective, only a few anti-leukocyte therapies have been directly validated in humans. Lack of tissue-tropism of targeted factors required for leukocyte accumulation and unspecific inhibition strategies remain the major challenges to ultimately translate therapies that modulate leukocytes accumulation into clinical practice. Here, we carefully describe receptor and ligand pairs that guide leukocyte accumulation into the atherosclerotic plaque and the infarcted myocardium, and comment on potential future medical therapies.

## Inflammatory Leukocyte Recruitment Promotes Cardiovascular Disease

Cardiovascular disease (CVD) represents the leading cause of mortality worldwide (Braunwald, 2012) and is mostly caused by atherosclerosis, a chronic inflammatory disease of middle- to large-sized arteries that is characterized by vessel-obstructing atherosclerotic plaques in the subendothelial space (Ross, 1999). The spontaneous rupture of atherosclerotic plaques, the subsequent formation of occlusive arterial thrombi, and the restriction of blood flow precipitates myocardial infarction (MI) and stroke (Minicucci et al., 2011). Initial atherosclerotic lesions develop in arteries with enhanced shear stress, turbulent blood flow, and endothelial dysfunction (Davignon and Ganz, 2004). This process is stimulated by traditional cardiovascular risk factors, such as smoking, hypertension, obesity, diabetes, and environmental stressors (Marchini et al., 2020). In atherosclerotic arteries, plasma low-density lipoproteins (LDL) are deposited in the subendothelial space and modified by oxidative processes. While oxidized LDL (oxLDL) exerts an inflammatory response of stromal cells itself, its uptake by tissue-resident macrophages initiates a myeloid-cell dominated pro-inflammatory cellular immune response (Swirski et al., 2007). It is now clear that inflammation is one of the key drivers of atherosclerosis, adverse cardiac remodeling, and myocardial scar formation after MI (Epelman et al., 2015). This response is characterized by the continuous accumulation of myeloid cells and lymphocytes in the atherosclerotic plaque, the myocardium, and draining lymph nodes of the heart (Epelman et al., 2015; Winkels et al., 2018; Farbehi et al., 2019; Wolf and Ley, 2019; Zernecke et al., 2020). Infiltrated leukocytes interact with stromal cells, secrete pro- or anti-inflammatory cytokines, and curb or promote inflammation and adverse tissue remodeling (Koltsova et al., 2012; Wolf et al., 2015; Sharma et al., 2020). While heart and vascular tissue contains small fractions of tissue-resident leukocytes that partially stem from embryonic origin (Wolf et al., 2015; Ensan et al., 2016), the recruitment and accumulation of blood-derived leukocytes represents a central and ongoing process that correlates with disease severity and clinical outcomes (Galkina et al., 2006; Swirski et al., 2006; Leistner et al., 2020). In addition, tissue inflammation promotes the local proliferation of macrophages and other leukocytes, although the relative contribution of *in situ* proliferation to the overall content of tissue leukocytes remains a matter of debate. While anti-leukocyte therapies are already in clinical use against Inflammatory Bowel Disease (IBD) and Multiple Sclerosis (Ley et al., 2016), it remains unknown whether similar strategies would be effective in cardiovascular pathologies. Here, we evaluate factors that promote leukocyte accumulation into the atherosclerotic plaque and cardiac tissue in mice and discuss their potential as targets for future medical therapies in CVD.

## Current Concept of Vascular Leukocyte Trafficking

The stepwise cascade of leukocyte recruitment comprises leukocyte rolling, chemokine-driven cell activation, integrin-dependent cellular arrest, and transmigration. This sequence of events has lately been refined by additional (and intermediate) states, such as slow rolling, adhesion strengthening, intraluminal crawling, paracellular and transcellular migration, and migration through the endothelial basement membrane (Ley et al., 2007). These processes in the leukocyte can be attributed to distinct classes and pairs of adhesion receptors and ligands: Initial rolling is mediated by the interaction of C-type lectins with glycoprotein ligands: E-Selectin on endothelial cells with leukocyte E-Selectin Ligand 1 (ESL-1) (Levinovitz et al., 1993) and endothelial P-Selectin and leukocyte L-Selectin with P-Selectin Glycoprotein Ligand 1 (PSGL-1) (McEver and Cummings, 1997). PSGL-1 is expressed on both, leukocytes (An et al., 2008) and endothelial cells (da Costa Martins et al., 2007). Integrins, α/β-heterodimers of a heterogeneous groups of 18 α- and 8 β-subunits (Takada et al., 2007), participate in (slow) rolling and mediate cell firm adhesion (Dunne et al., 2003). Of the 24 integrins, α_L_β_2_, α_M_β_2_, α_x_β_2_, α_d_β_2_, α_4_β_7_ and α_E_β_7_ are selectively expressed on leukocytes while α_2_β_1_, α_3_β_1_, α_5_β_1_, α_6_β_1_, α_6_β_4_, α_10_β_1_, α_v_β_3_ and α_v_β_5_ are expressed on ECs (Finney et al., 2017). Integrin-dependent leukocyte arrest is best established for the interaction of Very Late Antigen 4 (VLA-4, α_4_β_1_) with Vascular Cell Adhesion Protein 1 (VCAM-1) (Berlin et al., 1995; Ley and Huo, 2001), of Lymphocyte Function-associated Antigen 1 (LFA-1, CD11a/CD18, α_L_β_2_) with Intercellular Adhesion Molecule 1 (ICAM-1) (Meerschaert and Furie, 1995), and of Macrophage Receptor 1 (Mac-1, CD11b/CD18, α_M_β_2_) with EC-expressed ICAM-1 (Dunne et al., 2003) and CD40 ligand (CD40L) (Wolf et al., 2011, 2018; Michel et al., 2017). Firm adhesion is topically guided by the C-C motif chemokines CCL2 (Monocyte Chemoattractant Protein 1, MCP-1) and CCL5, and by the C-X-C motif chemokines CXCL1, CXCL4, and CXCL5 (Noels et al., 2019), which are secreted by cells in the atherosclerotic lesion or deposited by activated platelets (Drechsler et al., 2010) and subsequently presented on the glycocalyx (Graham et al., 2019). Binding of chemokines to their corresponding chemokine receptors on leukocytes, such as CCR2 (binding CCL2) or CCR5 (binding CCL3, −4, and −5), is critical for adhesion strengthening (Zernecke and Weber, 2014) and partially requires sialylation of CCRs by leukocyte-expressed α2,3-sialyltransferase IV (St3Gal4) as exemplified by CCR5 (Doring et al., 2014). Chemokine binding results in activation-dependent conformational changes in integrins (inside-out signaling) that induces an extended intermediate- and high-affinity structure of integrins (Arnaout et al., 2005; Fan and Ley, 2015; Fan et al., 2016) with a ∼10,000-fold increased affinity for their ligands (Shimaoka et al., 2003). Leukocyte migration is further supported by proinflammatory cytokines, such as IL-1β, that induce an upregulation of ICAMs, Platelet/Endothelial Cell Adhesion Molecule 1 (PECAM-1) (Mamdouh et al., 2003), and Junctional Adhesion Molecule A (JAM-A) (Martin-Padura et al., 1998). Transendothelial cell migration requires leukocyte integrins, in particular Mac-1 (Ley et al., 2007). While this cascade ultimately results in the accumulation of most leukocytes, a sub-population of Ly6C^low^ monocytes remains crawling on the endothelium for surveillance of endothelial integrity engaging LFA-1, C-X_3_-C Chemokine Receptor 1 (CX_3_CR1) (Auffray et al., 2007), and ICAM-1 and ICAM-2 (Ancuta et al., 2009). Whether these patrolling monocytes eventually transmigrate and contribute to the pool of tissue leukocytes remains a matter of debate (Auffray et al., 2007; Nahrendorf et al., 2007; Heidt et al., 2014a; Hilgendorf et al., 2014; Quintar et al., 2017).

**FIGURE 1:**
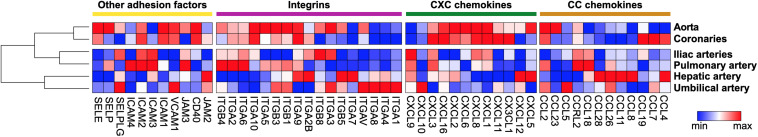
Gene expression pattern of adhesion factors expressed in human endothelial cells. Baseline gene expression of human endothelial cells from different locations was extracted from the curated gene set collection of the EndoDB database (Khan et al., 2019). Extracted expression values were plotted as heatmap by Morpheus with column minimum and maximum normalization. Within classes of adhesion receptors, rows and columns were sorted by hierarchical clustering.

The (numeric) regulation of leukocyte recruitment occurs via several mechanisms: First, leukocytes are activated by cytokines such as Tumor Necrosis Factor (TNF)-α or by oxLDL that promote expression of selectins (Stocker et al., 2000) and integrins (Couffinhal et al., 1994; Kita et al., 2001). Second, leukocyte activation may occur via an interaction with other cells, such as platelets that secrete leukocyte-activating factors as serotonin (Mauler et al., 2019). Third, the endothelium upregulates expression of adhesion receptors during systemic and local inflammation. Fourth, the pool of available leukocytes in the circulation is regulated by an enhanced production in the bone marrow or at sites of extramedullary hematopoiesis (EMH), such as the spleen (Swirski et al., 2009; Dutta et al., 2012; Heidt et al., 2014b). Under steady-state conditions, haematopoietic stem cell (HSC) homeostasis is regulated by bone marrow endothelial cell expressed CXCL12 (Stromal Cell-Derived Factor 1, SDF-1) that serves as retention and quiescence factor for HSCs and progenitor cells in the bone marrow niche that express its receptor CXCR4 (Mendez-Ferrer et al., 2008, 2010; Wolf and Ley, 2015; Krohn-Grimberghe et al., 2020). In the setting of inflammation, an enhanced sympathetic tone reduces CXCL12 expression in the bone marrow and increases CCL2 in bone marrow sinusoids that guides newly generated monocytes into the circulation (Krohn-Grimberghe et al., 2020). The migration factors required for seeding HSCs and progenitor cells to the sites of EMH are currently unknown. Fifth, tissue and cell tropism is regulated by a site-specific expression of adhesion factors: For instance, lymphocyte trafficking in the gut is predominantly facilitated by leukocyte α_4_β_7_ and α_E_β_7_ and endothelial Mucosal Addressin Cell Adhesion Molecule 1 (MAdCAM-1) (Briskin et al., 1993) and E-Cadherin (Higgins et al., 1998). In a secondary analysis of vascular adhesion receptors from the endothelial database EndoDB (Khan et al., 2019), we found a predominant expression of P- (*SELP*) and E-Selectin (*SELE*), integrin subunits α_3_ (*ITGA3*), α_5_ (*ITGA5*), α_9_ (*ITGA9*), α_10_ (*ITGA10*), β_1_ (*ITGB1*) and β_3_ (*ITGB3*), and VCAM-1 (*VCAM1*) and ICAM-1 (*I*CAM*1*) in endothelial cells from human coronary arteries and the aorta compared to other vascular beds, suggesting these may figure as potent mediators of cardiac leukocyte accumulation during inflammation (Figure 1).

## Inflammatory Leukocyte Recruitment in Atherosclerosis

**FIGURE 2:**
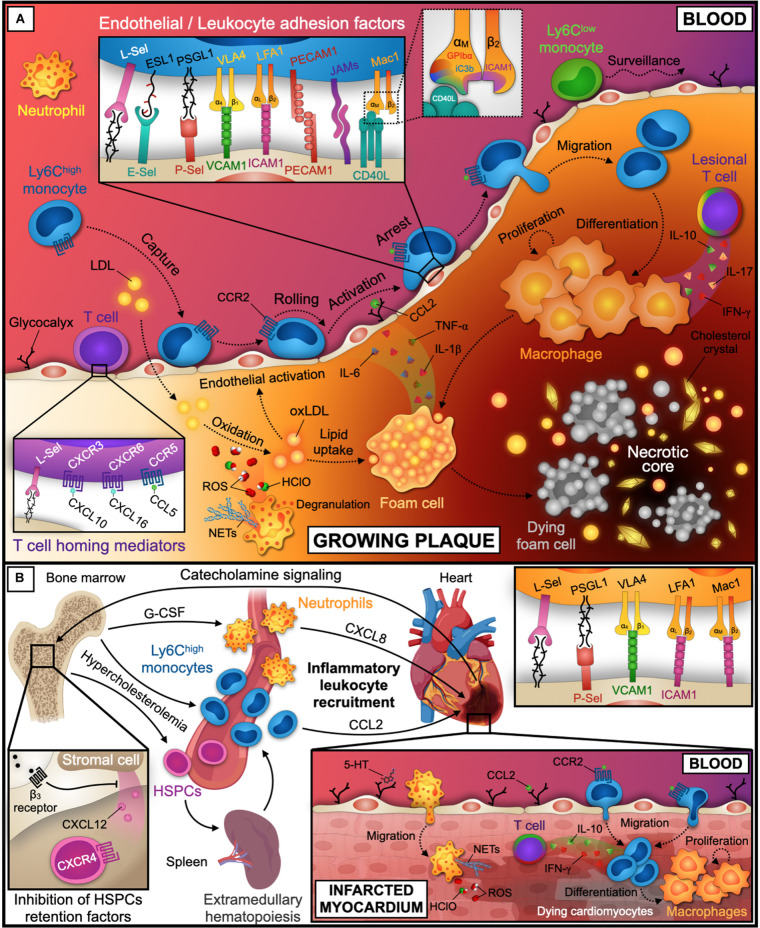
Leukocyte recruitment into the atherosclerotic plaque and infarcted tissue. **(A)** Initial endothelial dysfunction and activation is promoted by shear stress at sites of turbulent blood flow and lipid accumulation. While Ly6C^low^ monocytes patrol the endothelial surface for tissue surveillance, neutrophils and Ly6C^high^ monocytes are recruited into the subendothelial space. Within the plaque, Ly6C^high^ monocytes differentiate into macrophages. These proliferate, became foam cells, and orchestrate the inflammatory response, eventually die and build the necrotic core together with lipids and cholesterol crystals. These processes are further instructed by plaque-infiltrating T cells. Relevant inflammatory cytokines, chemokines, and receptor-ligand pairs for monocytes and T cells are indicated in the inlays. A third inlay shows CD40L binding to a distinct site within the I-domain of α_M_ chain of Mac-1 that does not interfere with other Mac-1 ligands. **(B)** Coronary artery occlusion precipitates MI and triggers progenitor and inflammatory leukocyte release from the bone marrow by adrenergic signaling and decreased expression of the retention factors CXCL12 and CXCR4 in the bone marrow niche. CXCL8 and CCL2 guide neutrophils and Ly6C^high^ monocytes to infarcted tissue. Neutrophils accumulate in the lesion by the adhesion factors depicted in the inlay and promote myocardial injury by reactive oxygen species (ROS), Hypochlorous acid (HClO), and NETs release. Ly6C^high^ monocytes are recruited and differentiate to macrophages. Tissue healing after MI is further modulated by infiltrated T cells that may secrete pro- or anti-inflammatory cytokines. LDL, low-density lipoprotein; oxLDL, oxidized LDL; Sel, Selectin; ESL1, E-Selectin Ligand 1; PSGL1, P-Selectin Glycoprotein Ligand 1; VCAM1, Vascular Cell Adhesion Molecule 1; VLA4, Very Late Antigen 4 (α_4_β_1_); ICAM1, Intercellular Adhesion Molecule 1; LFA1, Lymphocyte Function-associated Antigen 1 (CD11a/CD18, α_L_β_2_); Mac1, Macrophage Receptor 1 (CD11b/CD18, α_M_β_2_); PECAM1, Platelet/Endothelial Cell Adhesion Molecule 1; JAMs, Junctional Adhesion Molecules; CD40L, CD40 ligand; GPIbα, Platelet Glycoprotein Ibα; iC3b, inactive Complement component 3b; CCL, C-C Motif Chemokine Ligand; CXCL, C-X-C Motif Chemokine Ligand; CCR, C-C Motif Chemokine Receptor; CXCR, C-X-C Motif Chemokine Receptor; ROS, Reactive Oxygen Species; HClO, Hypochlorous acid; NETs, Neutrophil Extracellular Traps; IL, Interleukin; TNF, Tumor Necrosis Factor; INF, Interferon; G-CSF, Granulocyte Colony-Stimulating Factor; HSPCs, Hematopoietic Stem and Progenitor Cells. The figure was generated with schematics from BioRender.com.

A multitude of established receptor-ligand pairs has been validated mostly in experimental atherosclerosis in mice deficient for LDL-receptor (*Ldlr*^–/–^) and Apolipoprotein E (*Apoe*^–/–^), which exhibit diet-induced hypercholesterolemia (Wolf et al., 2015). Important recruitment factors include selectins, integrins, and other classes of adhesion factors (Galkina and Ley, 2007b) that can act in different cell types (Galkina and Ley, 2007a; Soehnlein, 2012; Gerhardt and Ley, 2015; Saigusa et al., 2020; Figure 2A):

### Cardiac Endothelial Cells

Endothelial cell expressed selectins (CD62) interact with glycoprotein ligands to mediate the capture and slow-down of circulating leukocytes. In humans, P-Selectin is not detectable in the healthy arterial endothelium but it is upregulated by oxLDL (Gebuhrer et al., 1995) and highly expressed in atherosclerotic lesions (Johnson-Tidey et al., 1994). Likewise, E-Selectin is detectable on the endothelium of human atherosclerotic plaques (Davies et al., 1993). While P-Selectin deficiency (*Psel*^–/–^) in *Apoe*^–/–^ and *Ldlr*^–/–^ mice (Johnson et al., 1997; Dong et al., 2000) neutralizes leukocyte trafficking and delays disease progression (Mayadas et al., 1993), *Apoe*^–/–^*Esel*^–/–^ mice are less affected (Collins et al., 2000). A combined deficiency of P- and E-Selectin in *Ldlr*^–/–^ mice abolishes atherosclerosis (Dong et al., 1998). P-Selectin, but not E-Selectin, expression correlates with human plaque stability (Tenaglia et al., 1997). Deficiency of VCAM-1 (Cybulsky et al., 2001) and ICAM-1 (Nageh et al., 1997) diminishes plaque size in mice. VCAM-1 is upregulated by proinflammatory cytokines at atherosclerosis-prone sites of arteries in *Apoe*^–/–^ and WT mice and mediates leukocyte arrest by binding to VLA-4 (Nakashima et al., 1998; Ley and Huo, 2001; Jongstra-Bilen et al., 2006). Leukocyte adhesion on the endothelium is also supported by binding of ICAMs to LFA-1 (Meerschaert and Furie, 1995) and of CD40L to Mac-1 (Zirlik et al., 2007). Small interfering RNAs (siRNAs) targeting multiple endothelial adhesion molecules reduced atherosclerosis in *Apoe*^–/–^ mice markedly (Sager et al., 2016a), while specific targeting of Mac-1 binding to CD40L by a peptide inhibitor (Wolf et al., 2011), or a blocking antibody (Wolf et al., 2018) prevented inflammatory leukocyte recruitment (Michel et al., 2017) in mice.

### Monocytes

In *Apoe*^–/–^ and *Ldlr*^–/–^ mice, hypercholesterolemia results in an expansion of monocyte progenitors and systemic monocytosis (Soehnlein et al., 2013; Rahman et al., 2017), likely by a modulation of reverse cholesterol transport in Hematopoietic Stem Progenitor Cells (HSPCs) (Yvan-Charvet et al., 2010; Murphy et al., 2011) and accelerated extramedullary hematopoiesis (Robbins et al., 2012). Several reports have identified increased adrenergic signaling, impaired quiescence and retention of HSPCs as hallmarks of this response (Dutta et al., 2012; Courties et al., 2015; Sager et al., 2016b). In the plaque, classical/inflammatory Ly-6C^high^ monocytes represent the main monocyte subset and give rise to vascular macrophages (Swirski et al., 2007). In mice, migration of Ly-6C^high^ monocytes is regulated by an interaction of P-Selectin/PSGL-1 (An et al., 2008), VLA-4/VCAM-1 (Huo et al., 2001), Mac-1/CD40L (Wolf et al., 2011), and of CCR1 and −5 with their corresponding ligands (Tacke et al., 2007; Combadiere et al., 2008; Soehnlein et al., 2013). Notably, CCR1- but not CCR5-deficiency seems to protect only from early atherosclerosis in *Apoe*^–/–^ mice on a WD for 4 weeks, suggesting temporal differences in CCR-dependent leukocyte recruitment. While one report has excluded a role for CCR2 in classical monocyte recruitment (Soehnlein et al., 2013), other studies have highlighted that monocyte migration into the plaque and circadian rhythms of monocyte counts in the circulation are largely regulated by the CCR2-CCL2 axis (Boring et al., 1998; Tacke et al., 2007; Combadiere et al., 2008; Winter et al., 2018). Consistently, siRNA targeting CCR2 reduced the accumulation of Ly-6C^high^ monocytes in the plaque and retards lesion progression in *Apoe*^–/–^ mice (Leuschner et al., 2011). Intraluminal crawling is regulated by the interaction of LFA-1 and Mac-1 with endothelial ICAMs (Schenkel et al., 2004). PECAM-1 and JAMs mediate transendothelial migration (Gerhardt and Ley, 2015).

### T Cells

T cells represent the most abundant leukocyte lineage in atherosclerotic lesions (Winkels et al., 2018; Fernandez et al., 2019) and orchestrate inflammation by a variety of T cell cytokines with pro- (TNF-α, IFN-γ, and IL-17) or anti- (IL-10) atherogenic functions (Tedgui and Mallat, 2006). A part of lesional T cells recognizes self-antigens in LDL and its core protein, Apolipoprotein B (Wolf and Ley, 2019; Wolf et al., 2020) and exhibits mixed phenotypes of proatherogenic IFN-γ secreting T_H_1 and IL-10 secreting regulatory T (T_reg_) cells. The contribution of other T_H_ cell subsets, CD8^+^, and γ/δ T cells is less clear (Saigusa et al., 2020). Naïve and central memory, but not activated, T cells express L-Selectin for rolling on high endothelial venules (HEVs) and homing to lymph nodes (Weninger et al., 2001; Ley and Kansas, 2004). CCR7 acts as an homing guidance for lymph node entry of T cells (Worbs and Forster, 2007). T cell homing to mouse atherosclerotic lesions involves L-Selectin (Galkina et al., 2006) and CCL5/CCR5 (Li et al., 2016), CXCL10/CXCR3 (Mach et al., 1999), and CXCL16/CXCR6 (Wuttge et al., 2004): Decreased plaque size has been observed in *Ccr5*^–/–^ (Braunersreuther et al., 2007), *Cxcr3*^–/–^ (Veillard et al., 2005), *Cxcl10*^–/–^ (Heller et al., 2006), and *Cxcr6*^–/–^ (Galkina et al., 2007) *Apoe*^–/–^ mice, which seems to be caused by reduced numbers of T_H_1 cells and increased T_reg_ numbers. Consistently, CCL5 (Braunersreuther et al., 2008), CCR5, and CXCR3 (van Wanrooij et al., 2005, 2008) antagonists are atheroprotective in mice. *Apoe*^–/–^ mice deficient for CCR1, an alternative receptor for CCL5 (Braunersreuther et al., 2007), and *Cxcl16*^–/–^ *Ldlr*^–/–^ mice (Aslanian and Charo, 2006) develop enhanced atherosclerosis. The role of CCR7 and its ligands CCL19 and CCL21, which are detectable in atherosclerotic lesions from *Apoe*^–/–^ mice and humans (Damas et al., 2007), has been controversial with contradictory findings (Luchtefeld et al., 2010; Wan et al., 2013). Many adhesion factors and chemokine receptors are expressed on myeloid cells and lymphocytes, which renders results from mice with whole-body genetic deficiencies difficult to interpret.

### Neutrophils

Hypercholesterolemia and inflammation promote the expression of Granulocyte Colony-Stimulating Factor (G-CSF) in the bone marrow, which triggers a release of neutrophils (Drechsler et al., 2010). Neutrophils adhere to the endothelium in a P- and E-Selectin (Eriksson et al., 2001), and β_2_/ICAM dependent manner (Soehnlein, 2012). Neutrophil adhesion also involves platelet-derived CCL5 and CCR1 as well as CCR5 and CXCR2 (Drechsler et al., 2010), and leukotriene B4 binding to its high-affinity receptor BLT1 (Houard et al., 2009). Neutrophils can be detected in early and rupture-prone atherosclerotic plaques in *Apoe*^–/–^ mice (Rotzius et al., 2010). Their depletion reduces atherosclerotic lesion size in *Apoe*^–/–^ mice (Drechsler et al., 2010). Lesional neutrophils correlate with disease progression (Drechsler et al., 2010), the release of reactive oxygen species (ROS) (Hosokawa et al., 2011), and the formation of neutrophil extracellular traps (NETs) in mice (Warnatsch et al., 2015; Folco et al., 2018). Neutrophils promote LDL oxidation (Podrez et al., 1999), favor monocyte recruitment (Zernecke et al., 2008), macrophage activation, and foam cell formation (Gombart et al., 2005). They may contribute to endothelial erosion and plaque destabilization by hypochlorous acid production from myeloperoxidase (MPO) (Naruko et al., 2002) and matrix-degrading proteases (MMPs) activity, such as MMP-9 (Leclercq et al., 2007; Soehnlein, 2012).

## Inflammatory Cell Recruitment After Myocardial Infarction (MI)

MI precipitates ischemic injury, cardiomyocyte death, and cardiac tissue remodeling and accelerates atherosclerosis by an activation of hematopoietic stem cells in the bone marrow niche and increased leukocyte production (Dutta et al., 2012; Figure 2B). In humans, neutrophils peak within the first 24 h after MI, likely by a G-CSF dependent response (Lieschke et al., 1994; Cannon et al., 2001; Zhang et al., 2015). Mouse neutrophils accumulate in the infarcted myocardium during the first 2 days after MI (Vafadarnejad et al., 2020) and contribute to ischemia/reperfusion injury by ROS release (Duilio et al., 2001), MPO activity (Askari et al., 2003), and NETs formation (Ge et al., 2015). Neutrophils are recruited by a process that involves CXCL8 (Sekido et al., 1993; Kukielka et al., 1995), platelet-derived serotonin (Mauler et al., 2019), L- (Ma et al., 1993) and P-Selectins (Weyrich et al., 1993), PSGL-1 (Hayward et al., 1999), β_2_ (CD18) integrins (Lu et al., 1997; Kempf et al., 2011), and ICAM-1 (Palazzo et al., 1998) in mice. While preclinical studies suggested that preventing neutrophil recruitment improves the clinical outcome after MI, anti-neutrophil therapy by blocking CD11b/CD18 has failed in clinical trials (Baran et al., 2001; Faxon et al., 2002). VLA4/VCAM-1 dependent migration (Bowden et al., 2002), a narrow therapeutic time window (Williams et al., 1994), and a potential interference with protective cell types mediating tissue reparation (Horckmans et al., 2017) may explain these negative results. Monocytes and macrophages represent the dominating hematopoietic cell types in the healthy and infarcted heart (Farbehi et al., 2019) and participate in tissue healing and inflammation. Peripheral monocytosis has been associated with impaired myocardial healing in humans (Maekawa et al., 2002; van der Laan et al., 2014). While monocyte depletion abolishes tissue regeneration (van Amerongen et al., 2007), hypercholesterolemia-induced Ly-6C^high^ monocytosis accelerates cardiac remodeling and the development of heart failure in *Apoe*^–/–^ mice (Panizzi et al., 2010). Ly-6C^high^ monocytes are recruited into the heart via CCR2 and CCL2, CCL7 (Kaikita et al., 2004; Dewald et al., 2005) as well as by VCAM1-depedent mechanisms (Nahrendorf et al., 2009). B cells in the infracted heart may serve as source of CCL7 (Zouggari et al., 2013). siRNA targeting CCR2 (Majmudar et al., 2013), bone marrow endothelial cell-expressed CCL2 (Krohn-Grimberghe et al., 2020), or endothelial adhesion molecules (Sager et al., 2016a) reduces Ly-6C^high^ monocyte accumulation in infarcted tissue in mice. Together with neutrophils, Ly-6C^high^ monocytes contribute to the phagocytosis of dead and dying cardiomyocytes and secrete extracellular matrix proteases and pro-inflammatory cytokines (Nahrendorf et al., 2007). While neutrophils do not persist in infarcted tissue (Dewald et al., 2004; Yan et al., 2013), monocytes continue to accumulate and give rise to early inflammatory macrophages (Nahrendorf, 2018). 5–10 days after MI, a second set of monocytes expressing Ly6C^low^ accumulate in a CX3CR1-dependent manner (Nahrendorf et al., 2007) but can also stem from Ly-6C^high^ in later tissue healing (Hilgendorf et al., 2014). Ly6C^low^ monocytes primarily involve in tissue healing and may be instructed by protective regulatory T (T_reg_) cell-derived IL-10 (Weirather et al., 2014) or pro-inflammatory T cell expressing IFN-γ (Yang et al., 2006). The role of other chemokine ligands highly expressed in the infarcted heart, such as CCL3 and CCL4, remains unclear (Frangogiannis and Entman, 2005). In addition to traditional cardiovascular risk factors, environmental stressors (e.g., air pollutants) enhance inflammatory leukocyte recruitment to the infarcted myocardium by an upregulation of endothelial ICAM-1 and VCAM-1, Mac-1 activation, and the release of pro-inflammatory cytokines from macrophages (Marchini et al., 2016).

## Clinical Translation and Concluding Remarks

The inflammatory nature of atherosclerosis and MI has been established by many clinical and pre-clinical studies (Libby, 2002). Several novel therapeutic concepts targeting inflammation and immunity have arisen from this work (Libby and Everett, 2019). Consequently, the inhibition of receptors and ligands involved in the generation, adhesion, and transmigration of leukocytes has revealed a great potential for anti-leukocyte therapies at the preclinical stage. In contrast, clinical evidence has remained on a premature stage. Clinical studies indicate that leukocyte counts (Madjid et al., 2004; Adamstein et al., 2021) correlate with the appearance of MI and clinical atherosclerosis. In addition, atherosclerotic plaque size (Stone et al., 2011) and the accumulation of some, specialized leukocyte subsets predict complicated disease (Fernandez et al., 2019). However, only a few clinical studies have directly tested anti-leukocyte therapies: Administration of the P-Selectin blocking antibody Inclacumab prevented myocardial damage after MI and a percutaneous coronary intervention (PCI) (Tardif et al., 2013; Stahli et al., 2016). A neutralization of MCP-1 (CCL2) with antibodies and gene therapy showed effective in the prevention of leukocyte recruitment in atherosclerotic vessels after PCI in primates (Horvath et al., 2002; Ohtani et al., 2004). Likewise, a depletion of monocytes by liposomal alendronate partially reduced stent restenosis (Banai et al., 2013). On the other hand, inhibition of the chemokine MCP-1 (CCL2) with the compound Bindarit failed to reduce coronary restenosis following PCI and had no effect on major cardiovascular events (Colombo et al., 2016). Administration of the CCR2 blocking antibody MLN1202 proved safety in individuals at a high atherosclerotic risk. A single nucleotide polymorphism at the MCP-1 promoter region was associated with reduced high-sensitivity C-reactive protein levels (Gilbert et al., 2011), but effects on atherosclerotic lesions or cardiovascular outcomes have not been evaluated in this study. Recently, the CCR5 antagonist Maraviroc was shown to reduce atherosclerosis progression in HIV patients (Francisci et al., 2019).

Several conceptual challenges render the direct translation into cardiovascular medical therapies difficult. A lack of tissue-tropism remains the leading limitation. In contrast to an inhibition of the integrins α_4_β_7_ and α_E_β_7_ during IBD (Ley et al., 2016), it is unclear which adhesion receptors specifically mediate leukocyte recruitment to atherosclerotic plaques or the heart. An unspecific inhibition of homing factors involved in host-defense, tissue healing, and regeneration is at the risk to induce severe side effects. This is best documented by β_2_-integrins such as Mac-1 and LFA-1 that mediates a variety of beneficial and pathogenic effects. A genetic mutation of the β_2_-subunit in humans causes the severe immunodeficiency Leukocyte-Adhesion Deficiency (LAD). In addition, small molecule β_2_-integrin inhibitors and antibodies have caused the potentially fatal complication, Progressive Multifocal Leukoencephalopathy (PML) that is likely caused by a reactivation of John Cunningham Virus (JCV) in the central nervous system (Berger and Houff, 2010). Recent preclinical studies suggest that this problem could be overcome by a ligand-specific inhibition, as demonstrated for the α-subunit of Mac-1 to specifically interfere with the binding of some ligands involved in the interaction with platelets or the endothelium, but not others (Ehlers et al., 2003; Wang et al., 2005; Wolf et al., 2011, 2018). In contrast, inhibition of platelet integrins has successfully been used in cardiovascular medicine for anti-thrombotic therapy (Ley et al., 2016). The widespread clinical application of tolerable and highly effective anti-chemokine (Mollica Poeta et al., 2019) and anti-integrin therapies (Raab-Westphal et al., 2017) in inflammatory disease and cancer, however, holds the potential of future clinical trials to combat cardiovascular pathologies.

## Author Contributions

All authors listed made a substantial, direct and intellectual contribution to this work, and approved it for publication.

## Conflict of Interest

DW holds patents on the inhibition of the leukocyte integrin Mac-1 by peptide mimetics and antibodies (EP 2444101 A1/EP 3 260 133 A1). The remaining authors declare that the research was conducted in the absence of any commercial or financial relationships that could be construed as a potential conflict of interest.
